# The effects of integrated care on professionals: a systematic review

**Published:** 2012-09-04

**Authors:** B Janse, I.N Fabbricotti, R Huijsman

**Affiliations:** Institute of Health Policy and Management, Erasmus University Rotterdam, The Netherlands; Institute of Health Policy and Management, Erasmus University Rotterdam, The Netherlands; MBA, Institute of Health Policy and Management – Erasmus University Rotterdam, The Netherlands

**Keywords:** integrated care, effects, professionals

## Abstract

**Background and aim:**

Traditional care is increasingly being replaced by integrated care models, which often implies changes for health professionals involved. However, although literature on integrated care is abundant, the primary focus is rarely on professionals. Consequently, it is not clear if and how they are affected by integrated care interventions. The aim of this study, therefore, is to provide a systematic review of the literature on the effect of integrated care on professionals.

**Methods:**

We included empirical peer-reviewed journal articles in English and Dutch that have been published between 1970 and 2010 and report effects of integrated care interventions on professionals. Four databases were searched using a variety of terms for integrated care, effects and professionals. Articles were reviewed by two researchers. References of included studies were checked for additional relevant articles. This process resulted in 56 articles. Outcomes of effects were categorized into several broad themes knowledge and skills, communication, relationships and satisfaction. Additional categories of effects are expected after further analysis of the results.

**Preliminary results and conclusions:**

Studies primarily focus on interventions within and between primary care and hospital care, involving mostly general practitioners, nurses and specialists. Interventions in these studies were heterogeneous. Integrated care interventions increase clinical knowledge and skills of professionals involved, as well as their knowledge of other professional’s roles and other services. These interventions also improve communication and relationships between professionals. They do not seem to affect the satisfaction of professionals with the care that is given, although it does increase their satisfaction with other services involved. A preliminary conclusion is that integrated care, indeed, does not leave professionals unaffected and that this influence seems primarily positive.

The poster presentation will further elaborate on these effects for professionals, the interventions used in relation to these effects, as well as the research methods and the scientific weight of these effects. Additional categories of effects will be described.

